# Human Milk Oligosaccharides, Growth, and Body Composition in Very Preterm Infants

**DOI:** 10.3390/nu16081200

**Published:** 2024-04-18

**Authors:** Margaret L. Ong, Sara Cherkerzian, Katherine A. Bell, Paige K. Berger, Annalee Furst, Kristija Sejane, Lars Bode, Mandy B. Belfort

**Affiliations:** 1 Department of Pediatrics, Division of Newborn Medicine, Brigham and Women’s Hospital, Boston, MA 02115, USA; 2 Department of Pediatrics, Larsson-Rosenquist Foundation Mother-Milk-Infant Center of Research Excellence (MOMI CORE), Human Milk Institute (HMI), University of California San Diego, La Jolla, CA 92093, USA

**Keywords:** preterm infants, human milk oligosaccharides, growth, anthropometrics, body composition, nutrition, human milk, bioactive factors

## Abstract

Human milk oligosaccharides (HMOs) are bioactive factors that benefit neonatal health, but little is known about effects on growth in very preterm infants (<32 weeks’ gestation). We aimed to quantify HMO concentrations in human milk fed to very preterm infants during the neonatal hospitalization and investigate associations of HMOs with infant size and body composition at term-equivalent age. In 82 human-milk-fed very preterm infants, we measured HMO concentrations at two time points. We measured anthropometrics and body composition with air displacement plethysmography at term-equivalent age. We calculated means of individual and total HMOs, constructed tertiles of mean HMO concentrations, and assessed differences in outcomes comparing infants in the highest and intermediate tertiles with the lowest tertile using linear mixed effects models, adjusted for potential confounders. The mean (SD) infant gestational age was 28.2 (2.2) weeks, and birthweight was 1063 (386) grams. Exposure to the highest (vs. lowest) tertile of HMO concentrations was not associated with anthropometric or body composition z-scores at term-corrected age. Exposure to the intermediate (vs. lowest) tertile of 3FL was associated with a greater head circumference z-score (0.61, 95% CI 0.15, 1.07). Overall, the results do not support that higher HMO intakes influence growth outcomes in this very preterm cohort.

## 1. Introduction

Fortified human milk is recommended for very preterm infants (<32 weeks’ gestational age [GA]). Given their physiologic immaturity and vulnerability to injury across multiple organ systems, these infants are at heightened risk for impaired physical growth compared with infants born full-term, which has adverse consequences for their neurodevelopment [1,2]. Among very preterm infants, human milk protects against infection [3], modulates inflammation and immune function [4], contributes to microbiome development [5], and predicts improved brain development and neurodevelopmental outcomes [6,7,8,9,10]. In addition to its nutrient components, human milk contains many non-nutrient bioactive factors that support physiological processes related to nutrient digestion and absorption and confers unique health benefits in the very preterm infant, including decreasing the risk for necrotizing enterocolitis (NEC) [11,12,13,14,15,16]. Understanding how bioactive factors influence health outcomes in this vulnerable population is critical to developing new diet-based prevention and treatment strategies.

Human milk oligosaccharides (HMOs) are one class of bioactive factors of increasing interest. HMOs are the third most abundant solid component of human milk, with highest concentrations in colostrum and decreasing concentrations as the milk matures. Most HMOs are found exclusively in human milk. HMO composition is largely influenced by maternal genetics. In particular, a mother’s secretor and Lewis statuses are defined by the presence or near absence of specific fucosyltransferases, which dictate the HMOs that can be synthesized [17,18]. Other maternal factors, including body mass index and environmental variables like season, also influence HMO composition, although to a lesser extent than genetic determinants [19,20]. HMO exposure is modifiable through supplementation or fortification. For example, individual HMOs are added to some commercially available full-term infant formulas, but currently no strategies or products exist for preterm infants [21].

Emerging evidence from full-term infant cohorts suggests that HMOs may be associated with physical growth during infancy. In previous studies of breastfed full-term infants, among secretors, higher 2′FL (2′fucosyllactose) was associated with greater weight and length measures and 3′SL (3′sialyllactose) with higher weight, while higher 6′SL (6′sialyllactose), LNnT (lacto-*N*-neotetraose), and HMO diversity were associated with lower weight and length measures during the first year of life [22,23]. HMOs also appear to influence body composition. In those studies, higher 2′FL and DSLNT (disialyllacto-*N*-tetraose) were associated with increased fat mass, whereas higher LNnT and greater HMO diversity correlated with lower fat mass and body fat percentage [23,24]. However, the findings are variable, and the results are not consistent across all studies. Moreover, formula-randomized controlled trials and real-world studies have not shown added growth benefits among those receiving supplementation with select HMOs [25,26,27,28].

Among preterm infants, more rapid growth during NICU hospitalization predicts improved long-term neurodevelopmental outcomes [29]. Yet, the extent to which HMOs influence physical growth in hospitalized very preterm infants is uncertain. To our knowledge, only two prior studies have assessed associations of HMO exposures with growth outcomes in preterm infants. Although both identified several individual HMO exposures associated with improved anthropometric growth, results were inconsistent; in addition, the studies were limited by an incomplete assessment of body composition (only one measured body composition and focused exclusively on fat-free mass but did not consider fat mass), limited gestational age inclusion (<28 weeks or 27–34 weeks rather than all very preterm infants), and the timing of HMO measurements [30,31].

The goals of this study were to quantify HMO concentrations in milk fed to hospitalized very preterm infants and to investigate associations of HMOs with anthropometric and body composition outcomes close to discharge at term-equivalent age. Given the relevance to potential supplementation strategies, we were primarily interested in determining whether HMOs in highest vs. lowest concentrations predicted greater weight, length, head circumference, and fat-free mass, as these growth outcomes predict better neurodevelopment in children born very preterm [32,33,34]. Based on the available literature, 2′FL, 3-fucosyllactose (3FL), 3′SL, 6′SL, LNnT, DSLNT, and total HMOs were our primary exposures of interest. However, given the overall lack of data in very preterm infants, we also planned exploratory analyses on all quantified HMOs.

## 2. Materials and Methods

### 2.1. Study Design and Population

We conducted a longitudinal cohort study leveraging previously collected human milk samples and data from very preterm infants hospitalized at Brigham and Women’s Hospital in an academic Level III NICU from 2015 to 2018 [35]. Of 126 infants originally enrolled, the present study included 82 infants meeting the following criteria: (1) gestational age < 32 weeks; (2) two human milk samples available for HMO analysis; (3) ≥1 outcome measure collected; and (4) primarily maternal milk fed (defined as ≥75% of total enteral intake on day of life 28). Of these 82 participants, 65 had data for body composition. The most common reason for missing body composition data was a continued need for respiratory support, which cannot be accommodated during the assessment. The Partners Human Research Committee reviewed and approved this study. Parents provided written informed consent for their infant(s) to participate.

In our NICU, we use written clinical practice guidelines (available upon request) to standardize parenteral and enteral provision of nutrition. The enteral feeding goal for very preterm infants is 150–160 mL/kg/day. Human milk is routinely fortified to 24 kcal/oz with a multi-component bovine-based liquid human milk fortifier.

### 2.2. Milk Collection and HMO Analysis

Details of milk sample collection procedures have been published [35]. Briefly, sample collection began when mothers were producing sufficient milk to meet infant requirements, typically within 1–2 weeks after birth. On weekdays, bedside nurses collected 3–5 mL samples of unfortified, hand-warmed, and gently mixed milk just prior to feeding the infant. Samples may have comprised fresh milk, frozen milk, or a combination. Almost all samples were maternal milk only (3 comprised a combination of maternal and donor milk, 1 comprised donor milk only). Overall, we designed our approach to sample milk that best represented the infant’s actual diet. Milk samples were stored at −80 °C and subsequently thawed and aliquoted for HMO analysis. For the present study, we identified two milk samples for each participant, one early (Time 1, as close as possible to the establishment of full enteral nutrition) and one late (Time 2, as close as possible to NICU discharge). We calculated the average of the two measures to estimate cumulative exposure to HMOs over the entire NICU hospitalization.

HMO analysis was performed by high-performance liquid chromatography at the University of California, San Diego as previously described [36]. In brief, maltose was added to each milk sample as an internal standard. HMO isolation was performed using solid-phase extraction, fluorescence labeling with 2-aminobenzamide, and subsequent high-performance liquid chromatography with fluorescence detection. Individual concentrations of 19 HMOs were quantified against standard response curves and the internal standard reference. Total HMO concentration was calculated as the sum of 19 measured HMOs. Simpson’s diversity index was calculated to represent diversity in HMO composition. Secretor status was determined by presence (versus near absence, <100 nmol/mL) of 2′FL.

Based on the available literature in preclinical models and full-term infant studies, we defined our primary HMO exposures of interest as concentrations (µg/mL) of six specific HMOs (2′FL, 3FL, 3′SL, 6′SL, LNnT, DSLNT) and total HMOs. We also assessed maternal secretor status as an HMO exposure and conducted exploratory analyses for the remaining quantified individual HMOs, HMO diversity index, HMO-bound sialic acid, HMO-bound fucose (nmol/mL), and total sialylated and fucosylated HMOs (µg/mL) to explore potential influences of fucose and sialic acid exposure from HMOs on outcomes.

### 2.3. Growth and Body Composition Outcomes

We assessed anthropometric body size at birth and term-equivalent age near NICU discharge. Bedside nurses weighed infants on calibrated digital scales to the nearest 1 g. Two NICU registered dietitians measured infant length with a recumbent length board to the nearest 0.1 cm and head circumference with a non-stretchable tape to the nearest 0.1 cm. We calculated z-scores of weight, length, and head circumference for sex and postmenstrual age (PMA) using references published by Fenton [37]. At term-equivalent age, for infants who were stable off respiratory support, we used air displacement plethysmography to assess body composition, including fat mass, fat-free mass, and body fat percentage (PEAPOD, COSMED, Concord, CA, USA). We calculated z-scores for body composition using published reference curves [38].

### 2.4. Statistical Analysis

We reported demographic and clinical characteristics as means and standard deviations (or medians and interquartile ranges (IQRs) for skewed distributions) for continuous variables and frequencies with percentages for categorical variables. We used repeated measures correlation [39] to assess the degree of correlation between measurements at Time 1 and Time 2 and tested for differences between median concentrations of each HMO at Time 1 and Time 2 using the Wilcoxon signed rank test given the non-normal distributions of most HMO variables. We calculated mean HMO concentrations of Time 1 and Time 2 measurements. Using these mean values, we divided the cohort into tertiles (high, intermediate, low) for analysis, with the low tertile used as the reference category. We estimated associations of HMO tertiles with anthropometric and body composition outcomes using linear mixed effects regression to account for intrafamilial correlation among multiple gestations. Adjusted models controlled for potential confounding determined a priori by GA at birth, birth size z-score (weight used for body composition analyses), PMA at outcome measurement, infant sex, and maternal age. Data were analyzed using the rmcorr (version 0.5.4) and nlme (version 3.1-160) packages for R statistical software (R Core Team, Vienna, Austria, 2022).

## 3. Results

Characteristics of mother–infant dyads (*n* = 82) are presented in Table 1. Mothers had a mean (SD) age of 32.0 (6.4) years at delivery, and 76% were classified as HMO secretors. Infants had a mean (SD) GA of 28.2 (2.2) weeks and birth weight of 1063 (386) g. HMO exposure at Time 1 was based on milk samples collected at mean (SD) 13.7 (7.1) days of life [30.2 (2.2) weeks’ PMA]. HMO exposure at Time 2 was based on milk samples collected at mean (SD) 62.8 (24.3) days of life [37.2 (2.8) weeks’ PMA].

**Figure 1:**
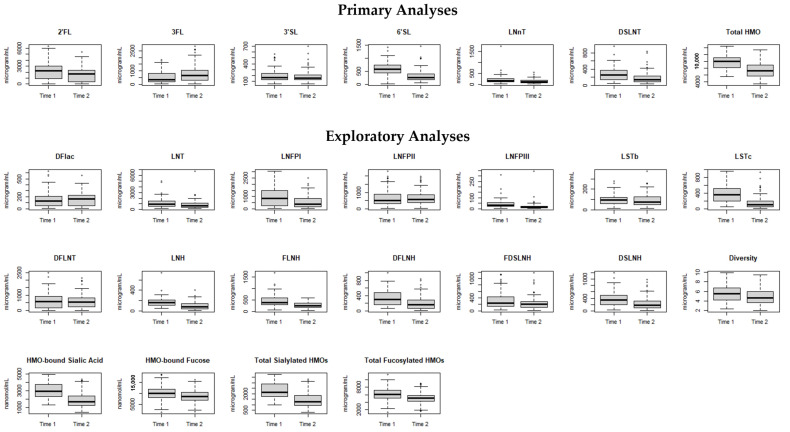
HMO exposures at two time points during NICU hospitalization among *n* = 82 very preterm infants fed predominantly maternal milk. Boxplots represent medians, interquartile ranges, and ranges with outliers (≥1.5 × IQR) notated as dots. Time 1 samples were taken as close as possible to the establishment of full enteral nutrition (13.7 (7.1) days of life [30.2 (2.2) weeks’ PMA]). Time 2 samples were collected as close as possible to NICU discharge (62.8 (24.3) days of life [37.2 (2.8) weeks’ PMA]).

When comparing the two time points, the concentration of almost all individual HMOs significantly decreased within individuals from Time 1 to Time 2 (Figure 1), except for 3FL, which increased [median (IQR) 352 (187, 783) vs. 638 (286, 1035) µg/mL, *p* < 0.01]. The repeated measures correlations between samples were mostly moderate to large, with r = 0.65 for 3FL and ranging from r = −0.37 to −0.75 for other HMO exposures (Supplemental Table S1). HMO exposures were categorized into tertiles based on the distribution of HMO concentrations in our cohort (Supplemental Table S2). We assessed term-equivalent outcomes at a mean (SD) of 39 (2) weeks’ PMA (Table 2).

For our primary HMOs of interest, exposure to mean HMO concentrations in the high (compared to low) tertiles was not associated with any of the anthropometric (Table 3) or body composition (Table 4) outcomes at term-equivalent age. We did observe an association of intermediate exposure compared to low exposure to 3FL with head circumference. Infants in the intermediate 3FL group had a 0.61 (95% CI: 0.15, 1.07) larger head circumference z-score at term-equivalent age than those in the low 3FL exposure group. Infants fed the highest concentration of 3FL also had a larger head circumference z-score (0.30, 95%CI: −0.15, 0.76) at term-equivalent age, but the confidence interval did not exclude the null.

Similarly, in exploratory analyses, being fed milk with the high tertile of mean HMO concentrations was not associated with higher anthropometric (Table 3) or body composition (Table 4) z-scores at term-equivalent age. Unexpectedly, we observed several significant associations of intermediate (vs. low) HMO exposures with length, head circumference, and body fat percentage. For example, infants exposed to the intermediate level of LS-tetrasaccharide c (LSTc) had a 0.48 (95% CI: −0.90, −0.05) lower length z-score compared to infants with low exposure, while infants with intermediate LS-tetrasaccharide b (LSTb) exposure had a 0.53 (95% CI: −0.99, −0.08) lower head circumference z-score than infants with low exposure. Additionally, intermediate lacto-*N*-tetraose (LNT) exposure was associated with a 0.73 (95% CI: −1.41, −0.06) lower body fat percentage z-score compared to low exposure. Maternal secretor status (comparing infants of secretor vs. non-secretor mothers) was not associated with anthropometric or body composition outcomes (Table 5).

## 4. Discussion

In this longitudinal cohort study of very preterm infants, we measured HMO concentrations at two time points spanning the NICU hospitalization. HMO concentrations varied substantially between participants and were moderately correlated within participants over repeated measurements. Most—but not all—HMO concentrations decreased modestly over time within individuals, consistent with prior studies [40,41]. Using the average value for each participant to reflect overall HMO exposures during the NICU hospitalization, we assessed the extent to which higher levels of specific HMOs were associated with a larger body size (weight, length, head circumference) and/or higher fat-free mass at term-equivalent age with the goal to identify future candidates for HMO supplementation or targeted pooling of donor human milk [42,43]. Overall, we did not observe significant associations of high (highest tertile) HMO exposures with a larger body size or composition at term-equivalent age compared with low HMO exposures, although the results suggest a possible association of higher 3FL with a larger head circumference.

There is a lack of data assessing the influence of HMO exposures on measures of physical growth among human-milk-fed preterm infants. While studies in full-term infants have identified certain associations with growth (positive associations of 2′FL and 3FL with weight, negative association of LNnT with weight and length), the results are inconsistent across studies [22,23,24,44]. Further, those findings may not generalize to the very preterm infant given the gastrointestinal immaturity, clinical illness, and overall vulnerability to growth impairment associated with prematurity. We identified only two prior studies of HMOs and physical growth among preterm infants. In one study of 106 extremely low-birth-weight (<1000 g) preterm infants, Wejryd et al. assessed anthropometric growth at 14 days, 28 days, and 36 weeks’ PMA in association with HMOs in maternal milk collected at day 14 [30]. Several individual HMOs were associated with higher anthropometric measurements in the first month of life, including LSTa with weight and head circumference and LNnT with head circumference; none of the associations persisted to 36 weeks’ PMA [30]. Another study by Roze et al. in 137 preterm infants found that 2′FL and total HMO were associated with greater linear growth and 6′SL with greater head circumference growth during NICU hospitalization; associations did not persist to the 2-year follow-up [31]. That study also assessed fat-free mass near discharge and found that 3′SL was associated with higher fat-free mass [31]. In contrast, we found that infants fed milk with intermediate levels of 3FL had higher head circumference z-scores at term-equivalent age compared to those with the lowest 3FL exposure; this finding is consistent with positive associations between 3FL and weight reported in studies of full-term infants [22,45,46] but was not identified in prior studies among preterm infants [30,31]. Notably, the other preterm studies and ours differed in GA; specifically, Wejyrd et al. included less mature infants (mean of 25 weeks at birth), whereas Roze et al. included more mature infants (31.3 ± 1.7 weeks at birth) [30,31]. It is possible that the effects of HMO exposure on growth may depend on GA at birth, which could explain differences in findings across studies. Moreover, the number of milk samples used to represent HMO exposure differed; Wejyrd et al. analyzed exposures at individual time points, while Roze et al. and our study utilized longitudinal HMO exposure averaged over repeated measures to capture exposure over time, which may be more robust to error. Modeling strategies also differed [30,31]. Notably, Wejyrd et al. reported unadjusted correlations without accounting for potential confounders [30], whereas Roze et al. adjusted for GA, birthweight z-score, infant sex, and days on antibiotics [31], and our study adjusted for GA, birth size z-score, infant sex, maternal age, and PMA at outcome. Taken together, findings are inconsistent across studies, which may be due in part to varying study design and analysis strategies. This also highlights the need for additional exploration with a longitudinal assessment of exposure and growth in larger preterm cohorts.

Although we did not observe any significant associations between high HMO exposure as compared with low HMO exposure and higher growth z-scores, in exploratory analyses, we unexpectedly found that, for certain HMOs, infants exposed to intermediate (vs. low) concentrations were smaller at term-equivalent age, suggesting slower growth. We observed that intermediate levels of LSTb and LSTc were associated with smaller head circumference and length z-scores, respectively. Associations of high levels compared with low levels of these HMOs were similarly in the negative direction, but their confidence intervals did not exclude the null. These results may be explained by chance, though are notably consistent with findings in the other preterm studies. Wejyrd et al. found that higher LSTb was associated with slower head growth and lower weight gain, and LSTc was associated with lower weight gain during NICU hospitalization [30]. While Roze et al. did not find associations with anthropometric growth, LSTc was associated with lower fat-free mass [31]. Although, we aimed to identify HMOs for which higher levels confer benefits to growth, our findings and other published findings identifying negative associations suggest potential benefits of exposure to lower (rather than higher) concentrations of specific HMOs for anthropometric outcomes.

In this study, HMO concentrations generally decreased over time during the NICU hospitalization, similar to other milk components such as protein [47], except for 3FL, which increased over time. The decrease in total and most individual HMOs and increase in 3FL are consistent with findings in most other preterm cohorts [30,48]. In contrast to our findings, Wejryd et al. observed a decrease in LNFPII and increase in LSTb and LNFPIII in extremely low-birth-weight infants [30]. Additionally, Roldan et al. found that 3FL and DFlac decreased, while several other fucosylated, sialylated, and neutral HMOs increased over time in very-low-birth-weight infants [49]. Overall, most HMO concentrations appear to decrease over time in preterm human milk. There is an overall similar pattern of decreasing concentrations in most HMOs over time among term infants [24,40]. Some differences have been observed between preterm and term milk for specific HMOs, including higher levels of specific sialylated HMOs (3′SL, DSLNT, LSTb, 6′SL, and LSTc) [48]. These predictable patterns of change over time may inform future supplementation or fortification strategies. Increasing concentrations of certain HMOs over time may hint at important biological functions at later stages of development but are not yet understood.

Although the mechanisms through which HMOs may influence growth remain largely unclear, the existing literature lends insight to potential hypotheses. Because HMOs are minimally digested and absorbed by the infant, effects may be linked to their primary prebiotic role within the gastrointestinal tract, shaping gut microbiota and subsequent nutrient accretion [40,46]. HMOs may also impact absorption by affecting intestinal barrier function [50,51]. Moreover, HMO activity may be indirect—through modulation of inflammation, which is detrimental to infant growth—or through other systemic effects mediated by the 1% of HMOs that are absorbed [4,20,52,53,54].

Our study has several strengths, including the repeated sampling of human milk and measurement of HMOs to represent cumulative exposure over time. We minimized measurement error in infant body length assessment by using a recumbent length board, and assessed the composition of body weight (fat mass, fat-free mass, body fat percentage) in addition to anthropometry. We measured and adjusted for several potential confounders, although residual confounding is a limitation of all observational studies. Our sample size limited our ability to detect small differences between groups and the ability to clearly elucidate non-linear relationships that may inform an optimal range for specific HMO concentrations. Chance associations are also possible given multiple comparisons. We assessed individual and total HMOs separately, but we acknowledge that interactions among HMOs as well as interactions of HMOs with other milk-borne bioactive factors as a biological system are possible [55,56].

## 5. Conclusions

In this cohort of very preterm infants, those exposed to the highest concentrations of individual and total HMOs did not differ in anthropometric or body composition outcomes at term-equivalent age compared to those with the lowest HMO exposures. Although specific HMOs may have other health benefits for very preterm infants, our results taken together with other studies do not support their individual current therapeutic potential to improve growth. Future work investigating the influence of HMOs on infant health should integrate the emerging concept of human milk as a biological system and consider HMOs’ interactions with each other and with other milk components.

## Figures and Tables

**Table 1:** Participant characteristics.

Maternal Characteristics (*n* = 76)	
Maternal age, years	32.0 (6.4)
Race	
Asian	5 (7%)
Black	23 (33%)
Multiple	3 (4%)
Other	3 (4%)
White	35 (51%)
Ethnicity—Hispanic	12 (16%)
Parity	1.6 (0.9)
Mode of delivery—Cesarean	50 (67%)
Infant Characteristics (*n* = 82)	
Gestational age, weeks	28.2 (2.2)
Male	46 (56%)
Multiple gestation	16 (20%)
Maternal milk at DOL 28—%	99 (4)
Postnatal steroid treatment	13 (16%)
NEC	2 (2%)
Late-onset sepsis	6 (8%)
IVH grade 3 or 4	3 (4%)
Infant Birth Measurements	
Weight (g)	1063 (386)
Weight z-score	−0.21 (1.05)
Length (cm)	36.2 (4.0)
Length z-score	−0.14 (1.22)
Head circumference (cm)	25.1 (2.7)
Head circumference z-score	−0.40 (1.10)

Data are represented as mean (SD) or *n* (%). Maternal milk at DOL 28 is represented as percentage of total enteral intake. NEC: necrotizing enterocolitis. IVH: intraventricular hemorrhage. Z-scores for anthropometric measurements were calculated based on reference curves published by Fenton [37].

**Table 2:** Anthropometric and body composition outcomes at term-equivalent age (39 ± 2 weeks postmenstrual age) for very preterm infants fed predominantly maternal milk.

Outcome	*n*	Raw	Z-Score
Weight (g)	82	2792 ± 610	−1.07 ± 1.30
Length (cm)	79	46.8 ± 2.9	−1.20 ± 1.36
Head circumference (cm)	78	33.4 ± 1.5	−0.47 ± 1.15
Fat mass (kg)	65	0.55 ± 0.21	1.79 ± 1.12
Fat-free mass (kg)	65	2.28 ± 0.46	−1.30 ± 1.56
Body fat percentage (%)	65	18.5 ± 4.7	2.23 ± 1.11

Data are represented as mean ± SD. Z-scores for anthropometric measurements were calculated using the reference published by Fenton [37]. Z-scores for body composition were calculated using the reference published by Norris [38].

**Table 3:** Adjusted associations of mean HMO concentrations in human milk (tertiles indicating high, intermediate, or low exposure) with anthropometric z-scores at term-equivalent age among primarily maternal-milk-fed very preterm infants.

HMO	Weight (*n* = 82)		Length (*n* = 73)		Head Circumference (*n* = 74)	
	High vs. Low	Intermediate vs. Low	High vs. Low	Intermediate vs. Low	High vs. Low	Intermediate vs. Low
Primary Analyses						
2′FL	−0.02 (−0.55, 0.51)	0.22 (−0.28, 0.73)	−0.11 (−0.54, 0.32)	0.10 (−0.32, 0.51)	−0.13 (−0.59, 0.33)	0.29 (−0.16, 0.73)
3FL	−0.04 (−0.57, 0.49)	0.05 (−0.48, 0.58)	0.12 (−0.32, 0.56)	0.25 (−0.20, 0.70)	0.30 (−0.15, 0.76)	**0.61** (0.15, 1.07) *
3′SL	0.04 (−0.47, 0.55)	−0.15 (−0.67, 0.38)	−0.25 (−0.67, 0.18)	−0.16 (−0.59, 0.26)	−0.03 (−0.48, 0.43)	0.10 (−0.37, 0.57)
6′SL	0.23 (−0.27, 0.73)	−0.29 (−0.77, 0.20)	−0.11 (−0.52, 0.31)	0.03 (−0.39, 0.45)	0.01 (−0.46, 0.47)	0.10 (−0.36, 0.55)
LNnT	−0.40 (−0.90, 0.10)	−0.45 (−0.94, 0.05)	−0.23 (−0.65, 0.19)	−0.19 (−0.60, 0.23)	−0.39 (−0.83, 0.05)	−0.01 (−0.46, 0.45)
DSLNT	−0.06 (−0.59, 0.46)	−0.32 (−0.84, 0.20)	−0.25 (−0.70, 0.20)	−0.18 (−0.61, 0.25)	−0.05 (−0.55, 0.44)	0.11 (−0.37, 0.60)
Total HMO	−0.04 (−0.56, 0.48)	−0.06 (−0.58, 0.47)	−0.14 (−0.56, 0.29)	−0.21 (−0.64, 0.22)	−0.13 (−0.60, 0.34)	0.01 (−0.47, 0.50)
Exploratory Analyses						
DFlac	−0.27 (−0.79, 0.26)	−0.09 (−0.61, 0.43)	−0.01 (−0.43, 0.41)	−0.07 (−0.50, 0.36)	0.20 (−0.27, 0.67)	0.27 (−0.21, 0.75)
LNT	0.08 (−0.44, 0.60)	0.002 (−0.51, 0.51)	0.12 (−0.30, 0.54)	0.21 (−0.22, 0.65)	0.05 (−0.42, 0.53)	0.15 (−0.31, 0.61)
LNFPI	−0.01 (−0.55, 0.54)	−0.01 (−0.53, 0.50)	−0.14 (−0.60, 0.32)	0.14 (−0.26, 0.54)	−0.23 (−0.72, 0.26)	0.07 (−0.38, 0.51)
LNFPII	0.20 (−0.32, 0.71)	0.11 (−0.41, 0.63)	0.07 (−0.35, 0.49)	−0.14 (−0.57, 0.29)	−0.004 (−0.44, 0.44)	0.38 (−0.09, 0.85)
LNFPIII	−0.01 (−0.56, 0.53)	−0.21 (−0.74, 0.31)	0.13 (−0.30, 0.56)	0.43 (−0.003, 0.86)	−0.02 (−0.52, 0.48)	0.07 (−0.40, 0.54)
LSTb	0.08 (−0.42, 0.59)	−0.42 (−0.93, 0.09)	−0.35 (−0.78, 0.08)	−0.17 (−0.59, 0.26)	−0.33 (−0.80, 0.14)	**−0.53** (−0.99, −0.08) *
LSTc	−0.03 (-0.55, 0.48)	−0.03 (−0.56, 0.50)	−0.15 (−0.56, 0.25)	**−0.48** (−0.90, −0.05) *	−0.33 (−0.78, 0.13)	−0.16 (−0.65, 0.33)
DFLNT	−0.19 (−0.71, 0.33)	−0.11 (−0.65, 0.43)	−0.09 (−0.54, 0.36)	−0.13 (−0.57, 0.30)	0.26 (−0.22, 0.74)	0.25 (−0.23, 0.72)
LNH	−0.21 (−0.72, 0.30)	−0.30 (−0.81, 0.21)	0.21 (−0.22, 0.64)	−0.04 (−0.49, 0.42)	−0.11 (−0.58, 0.37)	−0.01 (−0.48, 0.45)
FLNH	−0.03 (−0.57, 0.51)	−0.14 (−0.66, 0.38)	0.05 (−0.38, 0.48)	−0.21 (−0.64, 0.21)	−0.04 (−0.51, 0.43)	0.21 (−0.27, 0.69)
DFLNH	−0.09 (−0.61, 0.42)	−0.46 (−0.96, 0.03)	0.28 (−0.13, 0.70)	−0.13 (−0.53, 0.27)	0.06 (−0.41, 0.53)	0.20 (−0.27, 0.66)
FDSLNH	0.27 (−0.24, 0.77)	0.20 (−0.31, 0.71)	0.01 (−0.41, 0.43)	−0.26 (−0.69, 0.17)	0.19 (−0.26, 0.65)	−0.12 (−0.58, 0.35)
DSLNH	0.13 (−0.39, 0.65)	−0.31 (−0.81, 0.18)	0.01 (−0.42, 0.44)	0.01 (−0.42, 0.44)	0.11 (−0.34, 0.57)	−0.08 (−0.54, 0.39)
Diversity	0.23 (−0.28, 0.74)	0.16 (−0.35, 0.66)	0.07 −0.34, 0.49)	0.02 (−0.39, 0.43)	0.28 (−0.17, 0.73)	0.08 (−0.37, 0.54)
HMO-bound sialic acid	0.35 (−0.16, 0.87)	−0.25 (−0.75, 0.24)	−0.15 (−0.56, 0.27)	0.03 (−0.38, 0.44)	0.05 (−0.43, 0.53)	−0.15 (−0.61, 0.31)
HMO-bound fucose	−0.02 (−0.56, 0.52)	0.09 (−0.44, 0.62)	−0.07 (−0.50, 0.35)	−0.08 (−0.52, 0.36)	−0.20 (−0.66, 0.26)	−0.10 (−0.58, 0.38)
Total sialylated	0.36 (−0.17, 0.88)	−0.15 (−0.64, 0.35)	−0.10 (−0.51, 0.32)	0.02 (−0.39, 0.44)	0.09 (−0.39, 0.57)	−0.12 (−0.58, 0.34)
Total fucosylated	0.21 (−0.33, 0.75)	0.18 (−0.35, 0.71)	0.13 (−0.31, 0.58)	0.06 (−0.38, 0.51)	−0.11 (−0.58, 0.36)	0.02 (−0.44, 0.49)

Linear mixed effects models were adjusted for intrafamilial correlation among multiple gestations and controlled for potential confounding by gestational age at birth, birth size z-score, post-menstrual age at outcome measurement, infant sex, and maternal age. Beta estimates (95% confidence interval) indicate the z-score mean difference in outcome between the two indicated tertiles of HMO exposure. Individual, total sialylated, total fucosylated, and overall total HMOs were measured in µg/mL; HMO-bound sialic acid and fucose were measured in nmol/mL. Medians and interquartile ranges of HMO concentrations by tertiles are detailed in Supplemental Table S2. Bolded starred results represent findings for which the 95% confidence interval does not include the null (*p* < 0.05).

**Table 4:** Adjusted associations of mean HMO concentrations in human milk (tertiles indicating high, intermediate, or low exposure) with body composition z-scores at term-equivalent age among primarily maternal-milk-fed very preterm infants (*n* = 65).

HMO	Fat Mass		Fat-Free Mass		Body Fat Percentage	
	High vs. Low	Intermediate vs. Low	High vs. Low	Intermediate vs. Low	High vs. Low	Intermediate vs. Low
Primary Analyses						
2′FL	0.005 (−0.73, 0.74)	0.16 (−0.52, 0.83)	0.08 (−0.49, 0.64)	−0.01 (−0.54, 0.51)	0.14 (−0.62, 0.89)	0.11 (−0.59, 0.81)
3FL	0.18 (−0.59, 0.95)	0.45 (−0.25, 1.15)	0.10 (−0.50, 0.69)	0.30 (−0.24, 0.85)	0.09 (−0.72, 0.89)	0.29 (−0.44, 1.02)
3′SL	−0.07 (−0.75, 0.60)	−0.39 (−1.08, 0.31)	0.23 (−0.30, 0.75)	−0.12 (−0.66, 0.42)	−0.15 (−0.83, 0.54)	−0.53 (−1.25, 0.19)
6′SL	0.06 (−0.65, 0.78)	−0.26 (−0.93, 0.40)	0.03 (−0.53, 0.59)	0.01 (−0.51, 0.53)	−0.13 (−0.87, 0.61)	−0.37 (−1.06, 0.32)
LNnT	−0.64 (−1.31, 0.03)	−0.59 (−1.25, 0.06)	−0.03 (−0.58, 0.51)	−0.09 (−0.62, 0.44)	−0.66 (−1.36, 0.03)	−0.59 (−1.26, 0.07)
DSLNT	−0.53 (−1.24, 0.19)	−0.12 (−0.79, 0.56)	−0.27 (−0.83, 0.29)	−0.24 (−0.77, 0.29)	−0.55 (−1.27, 0.18)	−0.23 (−0.92, 0.46)
Total HMO	−0.21 (−0.92, 0.51)	0.10 (−0.58, 0.77)	−0.06 (−0.61, 0.49)	−0.13 (−0.66, 0.39)	−0.26 (−1.00, 0.47)	0.02 (−0.67, 0.72)
Exploratory Analyses						
DFlac	−0.21 (−0.90, 0.47)	−0.51 (−1.19, 0.18)	0.09 (−0.43, 0.60)	−0.44 (−0.96, 0.08)	−0.28 (−0.99, 0.42)	−0.51 (−1.22, 0.21)
LNT	−0.42 (−1.13, 0.29)	−0.41 (−1.09, 0.27)	−0.0001 (−0.56, 0.56)	−0.11 (−0.64, 0.43)	−0.63 (−1.34, 0.08)	**−0.73** (−1.41, −0.06) *
LNFPI	−0.32 (−1.07, 0.44)	0.20 (−0.46, 0.85)	−0.10 (−0.69, 0.50)	0.18 (−0.33, 0.69)	−0.25 (−1.04, 0.53)	0.16 (−0.53, 0.84)
LNFPII	−0.12 (−0.82, 0.58)	−0.15 (−0.86, 0.56)	0.10 (−0.44, 0.64)	0.25 (−0.30, 0.79)	−0.32 (−1.04, 0.40)	−0.47 (−1.18, 0.25)
LNFPIII	−0.06 (−0.80, 0.68)	−0.10 (−0.85, 0.64)	−0.17 (−0.74, 0.40)	−0.02 (−0.60, 0.55)	0.03 (−0.74, 0.79)	−0.07 (−0.84, 0.70)
LSTb	−0.29 (−0.98, 0.40)	−0.54 (−1.21, 0.13)	−0.44 (−0.97, 0.08)	−0.47 (−0.98, 0.04)	−0.18 (−0.90, 0.53)	−0.57 (−1.26, 0.12)
LSTc	−0.41 (−1.07, 0.24)	−0.43 (−1.12, 0.26)	−0.15 (−0.66, 0.36)	0.13 (−0.40, 0.67)	−0.45 (−1.13, 0.23)	−0.34 (−1.05, 0.36)
DFLNT	−0.33 (−1.03, 0.37)	0.19 (−0.56, 0.95)	−0.11 (−0.64, 0.43)	−0.50 (−1.08, 0.08)	−0.40 (−1.10, 0.31)	0.28 (−0.48, 1.05)
LNH	−0.27 (−0.98, 0.43)	−0.10 (−0.80, 0.59)	−0.06 (−0.61, 0.49)	0.02 (−0.52, 0.56)	−0.44 (−1.16, 0.29)	−0.37 (−1.07, 0.34)
FLNH	−0.01 (−0.76, 0.74)	−0.28 (−0.95, 0.39)	0.09 (−0.49, 0.67)	−0.23 (−0.74, 0.29)	−0.20 (−0.97, 0.57)	−0.39 (−1.06, 0.29)
DFLNH	0.37 (−0.36, 1.11)	0.12 (−0.52, 0.76)	0.07 (−0.51, 0.64)	−0.03 (−0.53, 0.47)	0.25 (−0.51, 1.02)	0.02 (−0.64, 0.69)
FDSLNH	0.04 (−0.64, 0.72)	0.03 (−0.66, 0.71)	0.28 (−0.24, 0.81)	0.03 (−0.50, 0.56)	−0.25 (−0.96, 0.45)	−0.13 (−0.84, 0.58)
DSLNH	0.11 (−0.62, 0.85)	−0.09 (−0.74, 0.56)	−0.31 (−0.88, 0.26)	−0.14 (−0.64, 0.35)	0.07 (−0.69, 0.83)	−0.23 (−0.90, 0.43)
Diversity	−0.38 (−1.07, 0.31)	−0.20 (−0.87, 0.47)	−0.16 (−0.68, 0.36)	0.27 (−0.23, 0.78)	−0.59 (−1.28, 0.11)	−0.50 (−1.18, 0.17)
HMO-bound sialic acid	−0.10 (−0.80, 0.60)	−0.29 (−0.93, 0.35)	0.01 (−0.53, 0.55)	−0.27 (−0.76, 0.22)	−0.27 (−0.98, 0.45)	−0.47 (−1.11, 0.17)
HMO-bound fucose	−0.01 (−0.71, 0.69)	0.13 (−0.57, 0.84)	0.11 (−0.43, 0.65)	0.04 (−0.51, 0.59)	0.001 (−0.72, 0.72)	0.11 (−0.62, 0.85)
Total sialylated	−0.08 (−0.77, 0.62)	−0.18 (−0.83, 0.48)	0.02 (−0.52, 0.55)	−0.20 (−0.70, 0.30)	−0.27 (−0.98, 0.44)	−0.38 (−1.04, 0.27)
Total fucosylated	−0.03 (−0.73, 0.67)	0.11 (−0.60, 0.81)	0.12 (−0.42, 0.65)	0.32 (−0.22, 0.86)	−0.12 (−0.84, 0.60)	−0.12 (−0.85, 0.60)

Linear mixed effects models were adjusted for intrafamilial correlation among multiple gestations and controlled for potential confounding by gestational age at birth, birth weight z-score, post-menstrual age at outcome measurement, infant sex, and maternal age. Beta estimates (95% confidence interval) indicate the z-score mean difference in outcome between the two indicated tertiles of HMO exposure. Individual, total sialylated, total fucosylated, and overall total HMOs were measured in µg/mL; HMO-bound sialic acid and fucose were measured in nmol/mL. Medians and interquartile ranges of HMO concentrations by tertiles are detailed in Supplemental Table S2. The bolded starred result represents a finding for which the 95% confidence interval does not include the null (*p* < 0.05).

**Table 5:** Adjusted associations of maternal secretor status with infant body size and composition z-scores at term-equivalent age for primarily maternal-milk-fed very preterm infants.

Outcome Z-Score	*n*	Beta Estimate (95% CI)
Weight	82	−0.14 (−0.64, 0.36)
Length	73	−0.06 (−0.46, 0.34)
Head Circumference	74	0.13 (−0.31, 0.58)
Fat Mass	65	−0.14 (−0.81, 0.53)
Fat-Free Mass	65	−0.04 (−0.56, 0.48)
Body Fat Percentage	65	−0.11 (−0.81, 0.59)

Beta estimates (95% confidence interval) indicate the z-score mean difference in outcome between infants born to secretor mothers compared to infants born to non-secretor mothers (reference). Linear mixed effects models were adjusted for intrafamilial correlation among multiple gestations and controlled for potential confounding by gestational age at birth, birth size z-score, post-menstrual age at outcome measurement, infant sex, and maternal age. Secretor status was determined during HMO analysis based on the presence of 2′FL.

## Data Availability

The data presented in this study are available from the corresponding author on reasonable request and with approval of the IRB. The data are not publicly available to maintain privacy of personally identifiable data for the participants.

## Supplementary Materials

The following supporting information can be downloaded at: https://www.mdpi.com/article/10.3390/nu16081200/s1, Table S1: Repeated measures correlations between HMO concentrations at Time 1 and Time 2 in very preterm infants fed predominantly maternal milk during NICU hospitalization; Table S2: Medians and interquartile ranges by tertiles for HMO exposures among *n* = 82 very preterm infants fed predominantly maternal milk.
