# Supplementary material for: Human Milk Oligosaccharides, Growth, and Body Composition in Very Preterm Infants
Source: Nutrients. 2024 Apr 18;16(8):1200. doi: 10.3390/nu16081200 (PMC11054505; doi:10.3390/nu16081200)
Supplement: Supplementary file 1 [file nutrients-16-01200-s001.zip › nutrients-2937432-supplementary.pdf]

Supplemental Table S1. Repeated measures correlations between HMO concentrations at Time 1 and Time 2 in very preterm infants fed predominantly maternal milk during NICU hospitalization

HMO Exposure	Rho	p-value
	Primary Analyses	
2′FL	-0.41	<0.001
3FL	0.65	<0.001
3′SL	-0.09	0.40
6′SL	-0.72	<0.001
LNnT	-0.36	<0.001
DSLNT	-0.51	<0.001
Total HMO	-0.66	<0.001
	Exploratory Analyses	
DFlac	-0.01	0.90
LNT	-0.42	<0.001
LNFPI	-0.58	<0.001
LNFPII	0.04	0.71
LNFPIII	-0.44	<0.001
LSTb	-0.13	0.24
LSTc	-0.72	<0.001
DFLNT	-0.15	0.16
LNH	-0.66	<0.001
FLNH	-0.62	<0.001
DFLNH	-0.57	<0.001
FDSLNH	-0.36	<0.001
DSLNH	-0.53	<0.001
Diversity	-0.43	<0.001
HMO-bound sialic acid	-0.74	<0.001
HMO-bound fucose	-0.37	<0.001
Total sialylated	-0.75	<0.001
Total fucosylated	-0.49	<0.001

Data represent repeated measures correlation coefficients (and corresponding p-values) between HMO concentrations measured at Time 1 and Time 2.

Supplemental Table S2. Medians and interquartile ranges by tertiles for HMO exposures among n = 82 very preterm infants fed predominantly maternal milk

HMO Exposure	Low (n=27-28)*	Intermediate (n=26-28)*	High (n=27-28)*
Primary Analyses			
2′FL	44 (28, 596)	2066 (1781, 2196)	2980 (2680, 3473)
3FL	166 (81, 233)	518 (432, 575)	1080 (842, 1550)
3′SL	116 (102, 127)	165 (158, 178)	243 (212, 345)
6′SL	285 (241, 297)	409 (378, 453)	629 (577, 757)
LNnT	77 (43, 92)	138 (113, 148)	234 (215, 303)
DSLNT	106 (77, 129)	192 (178, 221)	362 (326, 461)
Total HMO	6936 (6280, 7299)	8296 (8195, 8631)	10564 (9693, 11216)
Exploratoryh Analyses			
DFlac	4 (3-61)	152 (132, 171)	246 (214, 351)
LNT	430 (304, 541)	796 (694, 1034)	1441 (1310, 1822)
LNFPI	190 (140, 233)	656 (480, 749)	1276 (1100, 1846)
LNFPII	297 (189, 358)	535 (462, 634)	1049 (842, 1627)
LNFPIII	13 (8, 16)	27 (22, 29)	42 (39, 70)
LSTb	57 (42, 67)	83 (79, 95)	150 (122, 176)
LSTc	126 (94, 142)	235 (216, 266)	407 (370, 496)
DFLNT	55 (14, 190)	584 (461, 652)	1029 (589, 1439)
LNH	73 (59, 95)	123 (108, 133)	185 (172, 213)
FLNH	226 (198, 252)	336 (316, 361)	479 (425, 551)
DFLNH	207 (151, 227)	311 (284, 324)	435 (387, 530)
FDSLNH	125 (103, 146)	224 (205, 246)	433 (330, 606)
DSLNH	132 (78, 152)	269 (235, 321)	516 (390, 595)
Diversity	3.5 (3.0, 4.0)	5.0 (4.9, 5.3)	6.9 (6.2, 7.5)
HMO-bound sialic acid	1653 (1475, 1824)	2324 (2059, 2599)	3325 (3018, 3693)
HMO-bound fucose	6755 (5784, 7815)	9419 (8981, 9811)	12708 (11376, 14032)
Total sialylated	1260 (1114, 1348)	1669 (1546, 1951)	2535 (2256, 2846)
Total fucosylated	4589 (4180, 4780)	5568 (5253, 5725)	7458 (6569, 7800)

Data are represented as medians and interquartile ranges for HMO exposures operationalized as the mean concentration of the Time 1 and Time 2 measurements by infant. Individual, total sialylated, total fucosylated, and overall total HMOs were measured in μg/mL. HMO-bound sialic acid and fucose were measured in nmol/mL. *Numbers of participants in HMO-specific tertiles differ slightly due to ties.
